# In vitro fertilization (IVF) in mammals: epigenetic and developmental alterations. Scientific and bioethical implications for IVF in humans

**DOI:** 10.1186/s40659-015-0059-y

**Published:** 2015-12-18

**Authors:** Patricio Ventura-Juncá, Isabel Irarrázaval, Augusto J. Rolle, Juan I. Gutiérrez, Ricardo D. Moreno, Manuel J. Santos

**Affiliations:** Bioethical Center and Department of Pediatrics, Faculty of Medicine, Pontificia Universidad Católica de Chile, Santiago, Chile; Department of Physiology, Faculty of Biological Sciences, Pontificia Universidad Católica de Chile, Santiago, Chile; Department of Cellular and Molecular Biology, Faculty of Biological Sciences, Pontificia Universidad Católica de Chile, Santiago, Chile; Bioethics Center, Universidad Finis Terrae, Pedro de Valdivia 1509, Providencia, Región Metropolitana, 7501015 Santiago, Chile

**Keywords:** In vitro fertilization (IVF) in mammals, Epigenetics, Developmental alterations, Bioethical implications

## Abstract

The advent of in vitro fertilization (IVF) in animals and humans implies an extraordinary change in the environment where the beginning of a new organism takes place. In mammals fertilization occurs in the maternal oviduct, where there are unique conditions for guaranteeing the encounter of the gametes and the first stages of development of the embryo and thus its future. During this period a major epigenetic reprogramming takes place that is crucial for the normal fate of the embryo. This epigenetic reprogramming is very vulnerable to changes in environmental conditions such as the ones implied in IVF, including in vitro culture, nutrition, light, temperature, oxygen tension, embryo-maternal signaling, and the general absence of protection against foreign elements that could affect the stability of this process. The objective of this review is to update the impact of the various conditions inherent in the use of IVF on the epigenetic profile and outcomes of mammalian embryos, including superovulation, IVF technique, embryo culture and manipulation and absence of embryo-maternal signaling. It also covers the possible transgenerational inheritance of the epigenetic alterations associated with assisted reproductive technologies (ART), including its phenotypic consequences as is in the case of the large offspring syndrome (LOS). Finally, the important scientific and bioethical implications of the results found in animals are discussed in terms of the ART in humans.

## Introduction

Human beings have the capacity to modify the environment and in this way to influence the development and survival of animal species and human beings. In this perspective, one topic that has had increasing importance is the impact that modifications of the environment have on early stages of mammalian development, which are particularly vulnerable to environmental changes.

In 1999 Barker et al. described the relation of maternal malnutrition during pregnancy and the threat of developing certain diseases in adulthood [1]. Greater risk of coronary disease, hypertension, type two diabetes, metabolic syndrome and others have been described. This has been called the fetal origins of adult diseases or the Barker hypothesis [2, 3]. The transcendence of this discovery has been named with the initials DOHaD (Developmental Origins of Health and Disease) [4, 5]. Soon afterwards it was hypothesized that this effect could also occur in the pre-implantation embryo [6–8]. This was confirmed in several studies in rats that showed that malnutrition and hypoproteic diets administered only during the pre-implantation stage resulted in altered development such as low birth weight and abnormal blood pressure [9–12]. These findings were very important to stimulate research on the influence that assisted reproductive technologies (ART) could have on development and epigenetic reprogramming in the pre-implantation period, as these techniques imply great changes in the environment [13, 14]. The major concerns are related to the possible effects that ART may have on normal development in humans. It has been calculated that in developed countries 1–3 % of children are conceived using these techniques [15].

Fertilization of eutherian animals occurs in the maternal oviduct. This is the natural and unique environment to achieve the necessary requirements for embryo life and its early and late development. The embryo conceived in vitro is manipulated and cultured in very different conditions [11, 16].

Conrad Waddington highlighted many decades ago the relevance of the environment in development [17]. He emphasized the importance of studying the conditions that control development that mediate the interactions between genotype and phenotype. Genetics had discovered the laws of inheritance and had explained how different characters are transmitted from parents to offspring. But, Waddington underlined, that there wasn’t much knowledge about the mechanisms of development. He named this process Epigenetics, which is now understood as the conditions that control the expression of genes that are highly influenced by environment. A more precise definition is: “The study of changes in gene function that are mitotically and/or meiotically heritable and that do not entail a change in DNA sequence” [18]. This concept has broadened biological research in order to understand how this process can be altered by the environment and thus influence normal development and impact the etiology, susceptibility and onset of adult diseases [19–24]. Epigenetics has demonstrated that normal development does not depend only on a healthy genome.

There is an important epigenetic reprogramming during gametogenesis and the preimplantational period of the embryo, especially in imprinted genes, defined by their parental origin [25, 26]. This period has a sensible window to environmental changes which can alter the process of reprogramming, and thereby affect survival and the early and late development of mammal embryos [27, 28]. The discovery of the epigenetic mechanisms that control gene expression, at a molecular level, has been very useful to understand and detect when there has been an alteration at this level [20, 29]. Some of the mechanisms known today are: cytosine-adenine methylation; histone modifications and the control that different microRNA has on gene expression [24, 29, 30]. In the mid-1990s Sasaki et al., and Tremblay et al. showed that rats produced in vitro carried imprinted gene alterations, especially in the H19 paternal gene [31, 32]. Today we have a huge amount of information, mostly derived from animals, about the alterations that can occur in embryos produced in vitro because of the manipulation and artificial environmental conditions associated with these techniques [33].

**Fig. 1:**
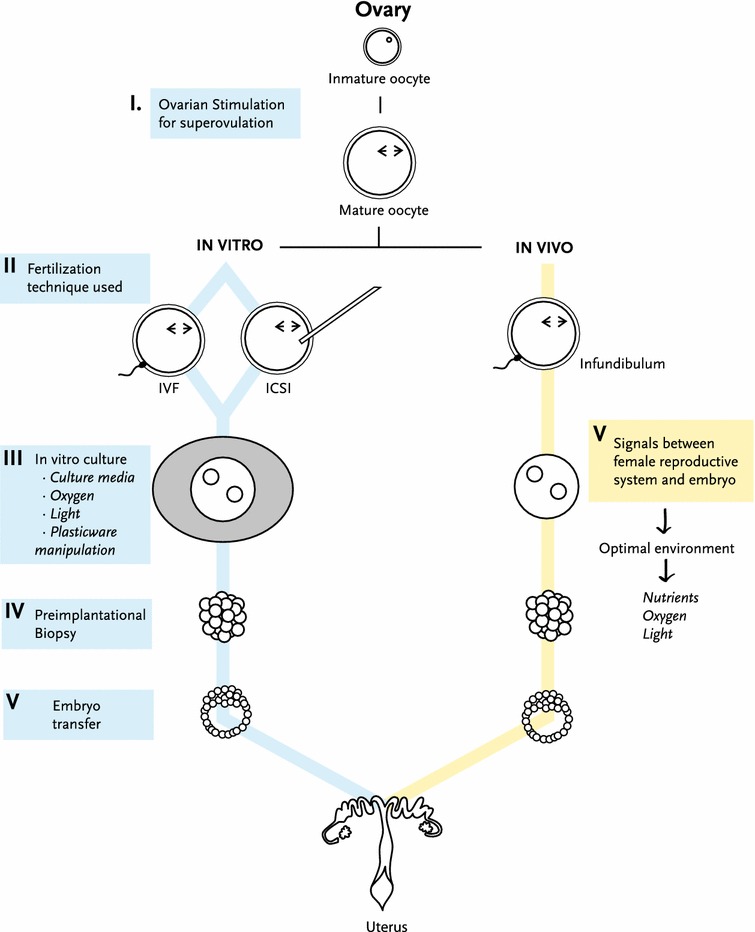
Schematic representation of in vivo and in vitro steps of mammalian fertilization. *I* Ovarian hormonal stimulation promotes follicle maturation and then ovulation. *II* ART uses different fertilization techniques to achieve fertilization, while in vivo female and male gametes interact and fuse in the female tract (infundibulum). *III* After fertilization, the preimplantation embryo spends a time under cultured conditions that may affect its further development. *IV* During this period of time, different techniques such as preimplantational embryo biopsies can be applied. *V* Finally, the in vitro produced embryo is transfered to a recipient female. On the other hand, the developing embryo moves towards the uterus interacting with the female reproductive system in a optimal environment

It has been discovered that the following processes and techniques associated with in vitro fertilization (IVF) can alter the epigenetic reprogramming of gametes, embryos and normal mammal development [34] (Fig. 1).

1. Superovulation and in vitro maturation (IVM) of gametes.
2. Fertilization technique: ICSI vs IVF.
3. Embryo culture.
4. Embryo manipulation: pre-implantation genetic diagnosis and embryo transfer.
5. Maternal-embryo signaling during the pre-implantation period.

The aim of this review is to update the currently available information provided by animal studies exploring offspring alterations in the epigenetic profile, development, survival and phenotype associated with the artificial environment in which IVF is performed. We propose that this information has scientific and bioethical implications that must be considered in human IVF/ICSI.

### Superovulation and IVM of gametes

#### Superovulation

In women the ovary normally produces one unique egg in each cycle. To count on more than one egg to perform IVF, the superovulation helps make the process more productive. This technique consists of hormonal stimulation of the ovary with exogenous gonadotropins or similar substances, to produce a larger number of eggs per cycle. The eggs obtained in this mode are usually immature and require an in vitro time of maturation.

Over the last 10 years there has been a growing body of evidence showing the relationship of superovulation and epigenetic disorders on eggs and embryos, which are associated with developmental alterations in different mammal species [22, 27, 35]. The analysis of the genomic imprinting of eggs obtained from super-ovulation shows disorders in four imprinted genes: Peg1, Kcnq1ot1, Zac and H19 in comparison with eggs from natural ovulation [36–40]. Specifically, alterations in gene H19 have been demonstrated in many studies [41, 42]. The hormone dose used for this procedure seems to be important in the degree of methylation [39].

Studies on naturally conceived versus super ovulation-derived mice offspring demonstrated epigenetic alterations in the somatic tissue of the developing embryo [25]. One recent study demonstrated that epigenetic alterations associated with gonadotropins also correlate with less fetal and placental development, as well as a smaller embryo throughout life [43]. Studies in mice suggest that ovary hyper-stimulation may affect embryo implantation [44].

This significant amount of information related to the deleterious effects of superovulation in normal gene expression, which impacts various issues necessary for embryo development [45, 46] has enhanced efforts to improve the superovulation protocols in animals to diminish its negative effects [47].

#### IVM of gametes

In vitro maturation of eggs has been associated with epigenetic alterations in addition to the effect of superovulation [48–50]. One study analyzed the DNA methylation status of the imprinted genes H19, Mest/Peg1 and Igf2R during in vitro maturation of mouse oocyte from preantral follicles [50]. The results show that when germinal vesicle oocyte after IVM are compared to those isolated from mice ovary, a loss of methylation at the Igf2R locus and Mest/Peg1 locus, and a gain of methylation at the H19 locus were found.

Similar results have been found in humans. In one of such studies, 20 metaphase II oocytes were analyzed, and 15 showed the normal unmethylated maternal pattern of H19 gene, while five originating from two different patients exhibited a methylated pattern [48].

It has been shown that the level of alteration depends on the time and the composition of culture media [39, 51–53].

Sperm culture has not been associated with epigenetic alterations, probably because epididymal sperm have the epigenetic reprogramming already completed, in contrast to egg maturation. The eventual epigenetic alterations that may be found in sperm have been associated with male infertility, as shown in mice [54, 55], swine [56] and humans [46, 57–59]. But a new challenge is presented when in animal and human ICSI is performed with immature sperm from the testes. Normal mice have been born with this technique [60]. After animal experiments, spermatozoa with different degrees of maturation have been used in humans with controversial results and low pregnancy rates. In vitro maturation of the sperm has also been tested. The in vitro culture of spermatids has also resulted in very poor outcome. Furthermore, if the sperm sample is maintained for an extended period of time there may be additional damage due to DNA fragmentation by nuclease release [61].

Egg epigenetic alterations associated with super-ovulation and in vitro culture can be maintained during the embryo and placental period [62, 63]. Authors recognize the difficulty in distinguishing the impact of super-ovulation versus the in vitro culture of eggs in these results.

In conclusion, there is evidence that superovulation as well as in vitro egg culture in different animal species produces epigenetic alterations in the egg and embryo, and that this could affect the outcome of the pregnancy.

### Technique of fertilization: ICSI vs IVF

There is evidence showing that the kind of IVF technique used can alter the epigenetic reprogramming and eventually development [64]. Although in regular IVF there is a selection of gametes and embryos of better quality, at present the prevalent and more economically efficient technique used in humans, is ICSI.

#### Intra-cytoplasmatic sperm injection (ICSI)

ICSI was introduced in 1992 with great success but without previous experimental testing. Animal models were considered unsuitable. This technique bypasses several physiological events: natural selection of the fertilizing sperm, sperm capacitation, acrosomic reaction and membrane fusion [65, 66]. The use of this technique in humans is increasing, and is used even as a standard method in cases with normal sperm [67]. Data provided from 56 reporting countries shows an increase of ICSI in all initiated cycles from 60.6 % in 2004 to 66 % in 2006 (96 % in the Middle East; 81 % in Latin America; 70 % in the USA) [68, 69]. The effects of ICSI in humans have been studied for decades, including implantation percentage, live-born, incidence of malformation and developmental disorders [70–73]. But since research specifically on epigenetic effects implies embryo destruction, it has not been performed in humans because of ethical reasons, underlining the importance of animal studies.

Studies on different animal species produced by ICSI, have found an asynchronous remodeling of chromatin decondensation of the male pronucleus in primates, [71, 74] mice and cattle [75, 76]. Mice produced by ICSI compared to those produced by regular IVF have long-lasting transcriptome disturbances that are maintained until the neonatal stage. But up to date these alterations have not correlated with changes in the phenotypic profile or with transgenerational effects [77]. It has been described that mouse ICSI blastocysts, compared to in vivo conceived groups, have a reduction in the inner mass cells and significant differences in gene expression related to cell function, development and metabolism [78]. However, there is one study that did not find any differences in pre-implantation development in IVF or ICSI-produced mice compared to naturally conceived mice. It is thought that the difference in the studies may be explained because of the protocol used for ICSI in different species and the different strategies used to activate the egg and guarantee embryo development [79, 80].

It has been observed in mice offspring produced by ICSI that DNA fragmentation is associated with more abnormal development, health and behavior [61]. That is why there is so much effort in selecting non-fragmented DNA sperm to have better results [81]. Finally, descendants of male mice produced by ICSI have a higher rate of apoptosis in their testicle sperm, which requires further research [82].

In conclusion, in animals, ICSI is a low efficiency technology and shows more epigenetic alterations in comparison with regular IVF. Thus its application in animals in contrast of what happens in human, is less widespread [80].

#### Plasticware exposure

Notably, in 2003 Hunt et al. [83] communicated that exposing female mice to the xenoestrogen Bisphenol A (BPA), meiosis was altered and the rate of aneuploidy increase in these animals. Other studies demonstrated that exposing mice to BPA at the implantation period produced alterations in uterine morphology, estrogen and progesterone receptors and a lower implantation percentage [84–87]. This compound, among other xenoestrogens, is used in plastic and epoxy resin production [88]. IMV, IVF, ICSI or embryo culture implies exposition to plasticware and different studies have shown leakage of xenoestrogens, phthalates and from plastic polymers [89]. Since many in vivo and in vitro studies have shown that these compounds induce epigenetic alterations such as cytosine methylation and/or histone acetylation [90, 91], the exposure of gametes and embryos to plasticware during in vitro manipulation may induce epigenetic abnormalities which will may affect further development.

This information motivated research on whether there are significant levels of chemical compounds in IVF, especially BPA, which could affect the reproductive process. In this review we did not find any animal studies addressing this aspect. There is one study on women that did not find detectable BPA either in the culture medium or in the material used for IVF [92].

### Embryo culture

#### *Culture media and nutrition*

In vitro culture (IVC) is probably the most relevant factor in the alterations of epigenetic reprogramming and development of animal embryos produced by IVF. This process is obligatory for IVF in contrast to some of the aspects mentioned above. Since the 1980s several studies have focused on investigating the effects of culture media in implantation, survival and development in different animal species, and the influence of several modifications in culture media such as the effects of proteins, the quality of the water and serum [93–99]. An increasing number of studies confirm the influence of the culture medium in epigenetic preimplantation reprogramming and its impact on early embryo development [100–102].

Various effects associated with in vitro embryo culture can be observed early in the period from fertilization to implantation, such as:

- Low implantation rate.
- Disturbances in development speed, embryo quality and low trophoblast development.
- Abnormal preimplantation epigenetic reprogramming.

It has been shown that suboptimal culture media affect the percentage of implantation and the survival of embryos that could achieve implantation [103]. It has been shown in cattle and other species that there is an association between the early timing of the first cleavage and the probability of reaching the blastocyst stage. This fact is thought to be related with the suboptimal IVC [104–106]. Gutierrez-Adan et al. also detected differences in the mRNA pattern and development speed between embryos produced in vitro and in vivo [107]. They hypothesized that this epigenetic pattern could also be a good system for the selection of better quality embryos [108, 109].

The suboptimal environment of IVC media also affects the development of the trophoblast [108]. In mice, the dysregulation of the trophoblast epigenetic profile is maintained in the placenta, which seems to be more sensitive than the embryo to IVC [110, 111].

The results of different studies clearly demonstrate that the above alterations are associated with different degrees of variation in the epigenetic profile of the embryo and placenta, and have an impact on development [112]. Of special importance are the modifications of imprinted genes such as gene H19, which has been widely studied because of the different expressions it has depending on the culture medium [32, 33, 47, 113, 114].

Later, after implantation various deleterious effects have been found in the fetal period (implantation to birth). These include:

- Unbalanced fetal-placental development
- Abnormal fetal growth
- Abnormal metabolic responses

In some cases these changes have been associated with epigenetic modifications in the preimplantation period, especially of imprinted genes that play a key role in fetal and placental development as has been indicated [115]. Recent studies have confirmed these findings, showing cellular aberrations in placenta and fetus linked to changes in gene expression, and the association of epigenetic aberrations with glucose metabolism and fetal growth in mice [116, 117]. This could affect the critical role that imprinted genes have in growth and development of the fetus and adult. Placental abnormalities and a greater mortality rate have also been reported [6].

The effect of impaired nutrition by maternal diet and in vitro culture is associated with developmental and metabolic alterations [118, 119]. Glucose metabolism is found to be altered in response to suboptimal culture conditions [120]. Specific alterations in glucose metabolism have also been also found to be different between male and female embryos [116]. Abnormal development of skeletal muscle in bovine fetuses associated with a decreased expression of mRNA for myostatin has been reported in embryos produced in vitro in comparison with in vivo [121]. These anomalies are not detected in the implantation period, but in later stages of development.

Effects in the postnatal and adult period have been described in relation to IVC. For decades it has been described that ruminants born by IVF have an abnormally large birth size and visceromegaly [122, 123]. The first and most relevant alteration in phenotype in animals produced by IVF is the Large Offspring Syndrome (LOS) [124]. It is characterized by large size at birth, gross abnormalities in different organs, mainly visceromegaly, and metabolic alterations, especially in the glucose-insulin system, hypoglycemia, large tongue and umbilical hernia. All these features are similar to those found in the Beckwith–Wiedemann (BWS) syndrome in humans [125]. It is of special interest that the epigenetic alterations in the LOS are very similar to those found in the BWS [126–128]. The phenotypic similarity of these syndromes has helped to understand the epigenetic alteration of the BWS. In fact one study has found in LOS the absence of methylation at the KvDMR1 on the maternal allele, which is the major molecular signature of BWS [125]. The relation between epigenetic defects in LOS and BWS is very complex and needs more study. Studies with the LOS bovine model seem to be a good approach [125].

Embryo culture itself, independent of the effects of embryo transfer and of fetal growth, is associated with higher systolic blood pressure in 21-week old mice compared to in vivo controls. This study also found elevated activity of serum angiotensin and hepatic enzymes involved in the control of gluconeogenesis [129].

The addition of serum to the embryo culture medium has been associated with abnormal skeleton and organ development [121]. In vitro culture of rat embryos results with little but significant alterations related to anxiety, psychomotor activity and special memory. Investigators propose that it is possible that a hippocampal alteration coupled to an impaired brain connection could alter memory development [130]. The type of culture medium also has a certain effect on the degree of these alterations in behavior [130]. Interestingly, a recent article reported that there is a synergistic effect of IVC with the type of diet given prenatally and postnatally in relation to its effect on anxiety and behavior. When these two parameters were evaluated separately there was no statistical difference. The authors point out that these findings highlight the importance of diet in women undergoing IVF [131].

The impact of culture medium on the outcome of ART is today undoubtedly a major constraint for these techniques in mammalian species. Thus special attention has been paid first to search for the best conditions of culture medium that can minimize its deleterious effects on epigenetic reprogramming and development [132–138]. And, secondly to research how to detect major alterations in the epigenetic profile of mammalian embryos produced by IVF [139–141]. In these latter aspects some authors have highlighted the importance of modifications in the expression of gene H19 that may be used as a sensor to the epigenetic embryo quality. Gene H19 has a central role in the control of imprinting genes [108]. Although there are differences between species, studies in animals may contribute to understand what happens in human [142, 143].

#### *Oxygen concentration effects*

Oxygen concentration in the oviduct where natural fertilization takes place in various mammalian species is between 1 and 9 %, which corresponds to an oxygen tension of around 11–60 mmHG [144–146]. In the beginning, human embryos produced by IVF were cultured at oxygen concentrations near 20 % (the same as the atmospheric level), very different than the in vivo condition [147, 148]. Studies in mice suggested that in vitro concentrations of oxygen similar to atmosphere levels could produce oxidative stress, mediated by free radicals of O_2_, and affect embryogenesis [149–152]. Today we have a large amount of data showing that culture of embryos at elevated O_2_ concentrations impairs blastocyst development, cell number and embryo metabolism in a variety of species [153–156]. Consistent with this, other studies have shown that culture at low oxygen pressure, around 5 %, help to produce better quality embryos in mice and cattle [157–160]. These deleterious effects were observed similarly during the cleavage and post-compaction phases [152]. Atmospheric oxygen concentration correlates with an increase of reactive oxygen species (ROS) compared to culture at 5 % oxygen [155]. ROS can alter protein synthesis and function and lipids, affecting cell membrane stability and DNA damage [161]. Experiments adding antioxidants to culture have had some effect in reducing oxidative stress [162–165].

Human studies have also shown the correlation between O_2_ pressures and IVF results [166, 167]. The Cochrane Data Base review confirmed the results of different studies in that the IVC of human embryos under conditions of low oxygen concentration improves the outcome of IVF and ICSI [168].

### Embryo manipulation: pre-implantation genetic diagnosis and embryo transfer

#### *Pre-implantation genetic diagnosis (PGD)*

This procedure has the aim to study the genetic condition of the embryo before implantation [169]. One or two blastomeres are extracted and genetically analyzed to perform this procedure.

There are studies in different species, especially cattle, to evaluate the effect of PGD [170]. PGD is performed in the embryo-transfer industry of animals for efficient sexing of the preimplantation bovine embryo in order to control the sex of the offspring [171]. The best technique to perform the biopsy with minimum damage to the embryo has been evaluated in mice and bovines [172, 173]. To assess the consequences of PGD on embryo and placental development a comparison between groups of biopsied mice versus non-biopsied control groups has been performed [174–176]. It was demonstrated with time-lapse videos that biopsied embryos had a delay from one embryo stage to another. This may probably be due to later compaction and hatching from the zona pellucida [177, 178].

The effects of blastomer biopsy on steroid metabolism have also been investigated in mice. The results demonstrated that mice born after this procedure had a lower birth weight and a deregulation of steroid metabolism, which could have severe effects on posterior development [175].

Recent studies that investigated short and long term effects on mouse development found behavioral disorders with three different tests (Morris, water maze and pole climbing tests), compared to control groups. These disorders may occur because of altered epigenetic patterns in the mouse brain [179–181].

#### *Embryo transfer*

Rivera et al. in a meticulous study isolated the effects of embryo transfer on mice independently of other factors [182]. To do this they studied the methylation profile of ten imprinted genes (H19, Snrpn, Igf2, Kcnq1ot1, Cdkn1c, Kcnq1, Mknr3, Ascl2, Zim1, Peg3). A control group of embryos was conceived in vivo, not cultured or transferred. The female mice were sacrificed on day 9.5 and the embryos immediately collected for epigenetic study (unmanipulated group). A second group, conceived in vivo, was extracted at the blastocyst stage and transferred after an hour and a half without passing through in vitro culture. Concepti were collected and processed as described in the control group (embryo transfer group). A third group of embryos conceived in vivo was extracted at the two blastomere stage and cultured in vitro for 2 or 3 days until blastocyst and then transferred. Concepti were collected and processed as described in the control group (embryo culture + transfer). The epigenetic profile was studied in the three groups at nine and a half days. The embryo transfer group conceived in vivo that was transferred without going through culture had an aberrant expression of imprinted genes compared to the control group. Furthermore, in the embryo cultures + transfer, the effects of transfer was increased by culture as shown by the number of genes with aberrant allelic expression in embryonic and extraembryonic tissues. Alterations in the imprinting pattern were more significant in the placenta and yolk sac. Interestingly, they found that biallelic expression of Kcnq1ot1 is related to loss of methylation on the maternal allele of the KvDMR1 locus, which has been often associated with the human syndrome Beckwith–Wiedemann (BWS). These data shows that the sole embryo manipulation induces aberrant methylation gene pattern.

### Maternal-embryo signaling during the pre-implantation period

In mammals, during the pre-implantation stage there is an exchange of different types of signals between the mother and the embryo that is thought to be critical for embryo development and implantation [183].

The presence of the embryo is recognized through these signals and a cross-talk takes place in the maternal tract which prepares an appropriate environment for implantation [184–186]. Thus, successful pregnancy in mammals involves synchronization between a receptive endometrium and a viable embryo [183, 187, 188]. This includes the modulation of the maternal immune system by the embryo [189, 190]. It is difficult to study these interactions in vivo; so much of what is known comes from in vitro models.

Absence of these signals in IVF raises the question of how much the technique can affect the results [191, 192]. In bovines it has been considered that the absence of these signals could be an important factor in the low efficiency of IVF [193]. Most recent reviews indicate that while there is considerable evidence of the influence of the oviduct on the quality of the developing embryo, there is little evidence of signals from the embryo to the oviduct or the endometrium in this early stage [109, 194–197].

By contrast, strong evidence exists in relation to the importance of the embryo/endometrium interaction for normal implantation and pregnancy development [198, 199]. Nevertheless new research has shown that the embryo has an effect on the regulation of the epithelial cells of the oviduct [200].

All this information addresses the fact of the key role of the oviduct and maternal embryo cross-talk in embryo development and implantation [201]. New genomic technologies open the horizon for future research in this aspect of pre-implantation development [184].

Several modifications to the technique to overcome this problem have been proposed, such as the use of intermediate host oviducts for IVF embryos or adding synthetic conditions similar to the oviduct liquid [202–204]. One vital factor of this exchange of signals for pre-implantation development is TGF- β [205].

The specific role of the environmental factors that we have discussed above in development and phenotype alterations are still not fully understood and difficult to isolate. The results of this artificial environment as a whole can be understood as the embryo being under different kinds of stress.

## Trans-generational inheritance

A transgenerational effect occurs when the alterations pass through several generations. Evidence shows that epigenetic imprinting could be trans-generationally transmitted, despite the fact that during gametogenesis there is epigenetic reprograming; but some molecular epigenetic elements not related to DNA sequence can resist this reprograming [206]. This has been studied in animals [206]. Some facts related to this “resistance” to epigenetic reprogramming are described in the large offspring syndrome (LOS), such as organomegaly, which could pass to a second generation of individuals [16]. In cryopreserved rabbit embryos it was demonstrated that IVF could alter female reproduction in the next generation [207]. A recent study in mice showed that suboptimal IVC was associated with transgenerational alterations of glucose metabolism and hepatomegaly in the male offspring [208].

Some relevant elements in epigenetic mechanisms related to this process are the following:

- Some specific types of transposons, which are resistant to post-fertilization demethylation, such as the intracisternal-A particles (IAPs,) long-terminal repeat retrotransposons [209].
- De novo mutations in the DNA sequence of preimplantation embryos such as: numerical and structural chromosomal abnormalities, point mutations, copy number variant (CNV) changes and duplications/deletions of microsatellites [210]
- Histone and chromatin modification [211]. Very recent data generated using *Caenorhabditis elegans* provide evidence for transmission of male gamete-mediated chromatin states through several rounds of replication, although it remains to be seen if a mechanism such as this is conserved in mammals [212].
- There are various cytoplasmic RNAs, such as nc(non-coding) RNAs which are transmitted by germ cells and are essential for post-fertilization development [213].

## Discussion

The general objective of animal reproduction research is mainly to improve breeding, reproductive and productive efficiency in harmony with ecological challenges and animal health [214]. For this purpose one of the essential topic is to study the genetic and epigenetic mechanisms that control the preimplantation embryo development in vivo and in vitro.

From another perspective it must be pointed out that animal studies are important to evaluate the safety of new drugs and treatments as a previous bioethical step before clinical trials or medical innovations are performed in humans [215].

The focus of this review was to update the scientific information concerning the epigenetic alterations produced by the techniques of in vitro manipulation related to IVF in animals and their impact in phenotype, behavior and transgenerational transmission. A substantial amount of research available in animals shows convincingly that ART particularly IVF, produces significant epigenetic modifications, altering the expression of different genes, particularly of imprinted genes that have a major role in normal animal development. The consequences of these perturbations on the outcome of the offspring raises important questions not completely resolved that need to be addressed in order to clarify them. On one hand, it is quite difficult to isolate the degree of influence of the different factors implicated in the ART technique, such as: Superovulation and IVC of gametes, ICSI, embryo culture and absence of maternal embryo-signaling. The existing information centers especially on the importance of embryo culture, which is essential to perform IVF. On the other hand, effects on the phenotype have been reported consistently in many studies and the finding of trans-generational effects in some studies is an interesting and important datum which needs more investigation. The most significant and clear fact is the LOS which mimics the BWS in humans.

These results obtained in animals rise scientific and bioethical questions of how this evidence should be considered on the increasing use of ART in humans [49]. Studies in animals, when possible and necessary are a bioethical imperative to assess the safety of new treatments and procedures; these are made before introducing the new techniques in medical praxis [216, 217]. However this process, as it is performed today, did not occur with human IVF, due to reasons that are beyond the scope of this review.

Although extrapolation of the results of animal experiments to humans has limitations due to differences among mammalian species, they frequently provide light on what may also happen in humans and are definitively valuable to orient new research.

Ideally, similar experiments as the ones performed in animals, could be done in humans and answer crucial questions. But there are major ethical restrictions to perform such epigenetic studies in human embryos. Most likely, in the near future the analysis of the epigenetic profile of children and adults born by IVF will be possible and this will allow access to information and consequences of this technique that at present are mostly unknown. We agree with several authors in terms that it is mandatory to have better human epidemiological studies and that efforts should be made in order to have a clinical follow up during the whole life of every child born by IVF, because epigenetic alterations in the early stages of development can be expressed later in adult life as different pathologies [218]. This is similar as the discoveries of Barker and others in relation to the effects environmental conditions of intrauterine life in pediatric and adulthood diseases.

Finally, we think that a major concern is how to give parents who seek IVF techniques a clear, objective and prudent information of the evolving knowledge of the possible risks involved in this procedure [219].
